# A flipped classroom, same-level peer-assisted learning approach to clinical skill teaching for medical students

**DOI:** 10.1371/journal.pone.0258926

**Published:** 2021-10-22

**Authors:** Enoch Chan, Michael George Botelho, Gordon Tin Chun Wong

**Affiliations:** 1 Li Ka Shing Faculty of Medicine, The University of Hong Kong, Pokfulam, Hong Kong SAR, China; 2 Division of Restorative Dental Sciences, Faculty of Dentistry, The University of Hong Kong, Pokfulam, Hong Kong SAR, China; 3 Department of Anaesthesiology, The University of Hong Kong, Pokfulam, Hong Kong SAR, China; King Saud University, SAUDI ARABIA

## Abstract

**Background:**

Clinical procedural skills are vital components of medical education. Increased student intake and limited capacity of medical schools necessitate more efficient ways to deliver clinical skill teaching. This study employed a flipped classroom, peer-assisted learning approach to deliver clinical skill teaching. It aimed to determine the influence of pre-class demonstration video watching and in-class student-student interactions on clinical skill acquisition.

**Methods:**

In 2017, a cohort of 205 medical students in their penultimate year of undergraduate medical study were recruited, and they learned bag mask ventilation and intravenous cannulation during this study. The participants watched a demonstration video before class, and then underwent self-directed practice as triads. Afterwards, each participant video-recorded their skill performance and completed post-class questionnaires. The videos were evaluated by two blinded assessors.

**Results:**

A hundred and thirty-one participants (63.9%) completed the questionnaire. For bag mask ventilation, participants who claimed to have watched the corresponding demonstration video before class achieved higher performance scores (those who watched before class: 7.8 ± 1.0; those who did not: 6.3 ± 1.7; *p* < 0.01). For intravenous cannulation, while there is no significant difference in performance scores (those who watched before class: 14.3 ± 1.3; those who did not: 14.1 ± 1.4; *p* = 0.295), those who watched the video before class received less interventions from their peers during triad practice (those who watched before class: 2.9 ± 1.8; those who did not: 4.3 ± 2.9; *p* < 0.05). The questionnaire results showed that most participants preferred the new approach of clinical skill teaching and perceived it to be useful for skill acquisition.

**Conclusion:**

The flipped classroom, same-level peer-assisted learning model is potentially an effective way to address the current challenges and improve the efficiency of clinical procedural skill teaching in medical schools.

## Introduction

Medical and health care students are required to master clinical procedures such as cannulation, ventilation support, physical examination and more, in order to become medical and health care professionals who can competently carry out these procedures in various clinical settings [1]. The archaic adage of “see one, do one, teach one” had been the traditional way of clinical training through observing real-life patient care [2, 3]. However, this approach is insufficient for modern days due to the increasing complexity of clinical procedures, in which case not all individuals can safely perform a procedure after observing only once [4–6].

Both hands-on physical practice and observational practice are indispensable for clinical skill acquisition. While observational practice is inferior to hands-on physical practice in terms of helping learners to gain proficiency in carrying out a procedure, it allows learners to process information including coordination patterns, subtle movements and possible difficulties in performing the procedure. Processing such information during concomitant hands-on practices is difficult [7, 8]. Many providers of medical and health care education substantially increased their student intake in order to address the shortage of health care workers, and this vastly increased the demand for individual opportunities to observe and practice procedural skills [9–11]. Given the already limited capacity in medical school campuses and teaching hospitals worldwide, this increased demand is not met. Studies across multiple medical schools in the United States showed a trend of medical students being underexposed to essential clinical procedures during their studies and feeling uncomfortable with performing these procedures [12–15]. These phenomena highlight the necessity of more efficient ways to ensure sufficient observational practice and physical practice opportunities in clinical skill teaching.

Dyad practice is useful for facilitating students’ alternation between physical practice and observation practice in clinical skill learning [8]. Some studies showed that dyad practice improved students’ skill performance in clinical skill training [16] as well as their ability to apply the skills learnt in novel settings [17]. Some studies also looked at medical or nursing students’ perception about dyad practices, and it was generally perceived that dyad practice facilitated information sharing, cross-checking, observing and getting support from peers. Students also felt more confident in performing the task and applying the skills learnt in real work environment [11, 18]. The participants from both of these studies also expressed their concerns about reduced opportunity for individual hands-on practice in the dyad practice setting [11, 18], but another study found that with the same duration, dyad practice was non-inferior to individual practice [19]. Furthermore, earlier studies on non-clinical procedural skills reported that practicing as triads and tetrads led to similar outcome as individual and dyad practices [20], but it is unclear if this principle applies to clinical skill learning as well.

The use of demonstration videos with exemplar or flawless demonstrations by clinical experts can promote the accessibility of observational practice in clinical skill teaching [21, 22]. Multiple studies showed that a blended learning approach combining demonstration videos with face-to-face teaching enhanced medical and nursing students’ knowledge and performance of clinical procedural skills. This approach was generally well received by students because the video resources allowed repeated viewing and flexibility [23–35]. A comparative study reported that medical students who had access to demonstration videos for physical examination achieved better results in objective structured clinical examination, compared to the cohort that did not have access [36]. However, some studies showed that while video exemplars as supplementary learning resources improved nursing students’ self-rated confidence and understanding, they made no significant impact on their knowledge and skills in performing clinical procedures [37, 38]. Some studies also showed that video-based learning was non-inferior to bedside tutorials and didactic lectures in terms of training clinical procedural skills [39, 40].

In this study, we devised a new model of clinical skill teaching for medical students through amalgamating flipped classroom and same-level peer-assisted learning. Many previous studies focused on whether providing demonstration videos enhances clinical skill acquisition in comparison to traditional approaches [24, 25], but they did not report if the timing of video watching influenced skill acquisition. Therefore, the present study aimed to determine whether the timing of video watching (i.e., pre-class versus in-class) would influence students’ clinical skill acquisition, through having a proportion of participants being given access to the demonstration video prior to the practical class, while the others were given opportunity to watch the demonstration video in class. During the practical class, the participants were divided into triads for self-directed practice.

The triad practice approach was designed based on a same-level peer dyad model previously reported in multiple studies [8, 16, 17, 19] and an earlier study which showed that triad practice was non-inferior to dyad practice for non-clinical procedural skill acquisition [20]. Each of the triads consisted of a “performer”, a “coach” and a “monitor”. The performer underwent hands-on practice, the coach observed and offered just-in-time feedback and hints to the performer, and the monitor observed and assessed the performer’s skill performance and the quality of feedback and hints given by the coach. While previous studies demonstrated the effectiveness of dyad practice in the context of clinical skill acquisition [8, 16, 17, 19], they did not study the effect of peer interaction per se. Therefore, in addition to measuring students’ skill performance after practicing as triads, this study also monitored student-student interaction and behavior during triad practices.

## Methods

### Ethical approval

This study was reviewed and approved by the Institutional Review Board of the University of Hong Kong / Hospital Authority Hong Kong West Cluster (UW 17–243). All participants provided informed consent to participate in this study.

### Participants

We recruited 205 of 5^th^-year students in the 6-year undergraduate Medicine program at The University of Hong Kong. The study took place between 1 August 2017 and 5 December 2017, and our participants were scheduled to learn bag mask ventilation (BMV) and intravenous cannulation (IVC) procedures at that time.

### Learning materials

The demonstration videos were developed by GTCW in consultation with other clinicians and disseminated to the participants via learning management system. The demonstration video for BMV was 6 minutes and 24 seconds long. From 0:00 to 1:14, the essential equipment required for BMV was introduced. From 1:15 to 2:44, the procedures for assembling and checking the self-inflating bag mask device were demonstrated. From 2:45 to 6:24, using a combination of video footage, still images, voiceover narration and annotations, the BMV procedure was broken down. Each key step was explained in detail, and the common mistakes were highlighted. It also highlighted issues that may arise during BMV and the techniques for troubleshooting.

The demonstration video for IVC was 7 minutes and 13 seconds long. From 0:00 to 0:34, the equipment required for IVC was introduced. From 0:35 to 5:40, using a combination of video footage, still images, voiceover narration and annotations, the IVC procedure was broken down into key steps, and each of them was explained in detail while highlighting the common mistakes. From 5:40 to 7:13, the IVC procedure was demonstrated once again without being broken down to key steps, and text annotations were added to signpost the key steps.

### Lesson rundown

The face-to-face clinical skill teaching sessions were 3 hours long. Each session was moderated by one tutor and accommodated 10 to 12 students, and all students practiced in a triad at least once. One half of the teaching sessions covered BMV before IVC (BMV^1st^ group), and the other half covered IVC first (IVC^1st^ group). The students were informed of the teaching arrangement via email at least 5 days before the face-to-face teaching sessions. Students of BMV^1st^ group were instructed to watch the demonstration video for BMV before class; and for the IVC^1st^ group, the demonstration video for IVC.

At the start of the teaching sessions, students were randomly assigned into triads. Both the coach and monitor were given access to the laminated handouts (S1 and S2 Files), the demonstration videos and the assessment rubrics (S3 and S4 Files) for quick reference while the performer was practicing. All students were given time to watch the assigned demonstration video prior the triad practice in case they did not do so before class. The tutor observed each triad and answered any questions that were raised, but did not offer hands-on demonstrations, so that all observational practices were afforded by either watching the demonstration videos or observing same-level peers’ performance of the skills.

During the triad practice, each member was given 10 minutes to fulfil their assigned role (performer, coach, or monitor). Then, the roles were rotated for two subsequent 10-minute segments that followed. All instances of referring to the demonstration video was recorded. Also, whenever the performer was given feedback or hints, it was counted as one intervention. Afterwards, each student took turn and performed the procedural skill without peer support. Video recording of the performance was carried out by their peers using mobile devices, without including the performer’s facial features.

The students were then randomly reassigned into new triads to learn the second clinical procedure, and they were instructed to watch the demonstration video which they did not have access before class. This was followed by 3 rounds of practice as triads, after which the video recording of skill performance was made.

### Assessment of competency

The video recordings of each student’s performance of BMV and IVC were independently assessed by two blinded assessors (senior anesthesiologists) using the assessment rubric (S3 and S4 Files). The final score for BMV and IVC performance was a weighted sum of all scores, where the weighting was agreed upon by the assessors based on the relative importance of each key step to the entire procedure.

### Student feedback

At the end of the session, the students were invited to complete a questionnaire (S5 File). The questionnaire was piloted with students during the first month of this study and was subsequently modified. Internal reliability of the questionnaire was determined by Cronbach’s alpha and item-to-total correlations.

### Statistical analysis

The number of interventions received and the performance scores given to each student were tested for normality using the Shapiro-Wilk test. Inter-assessor consistency in scoring was determined by Kendall’s tau-b and Wilcoxon signed-rank test. Comparison between subsets of students were carried out by Mann-Whitney U test or Pearson Chi-square test. Bivariate correlations between Likert scale items of the questionnaire were determined by Kendall’s tau-b. All statistical analyses were performed using IBM SPSS 25.0 (Armonk, NY).

## Results

### Students’ interaction in triads and clinical skill performance

Among the 205 students recruited in this study, 165 questionnaires were received. There were 131 students (79.4%) who claimed they have watched the video before class, while 18 students (10.9%) reported they did not. There were also 15 students (9.7%) who did not answer that question.

Table 1 presents the number of interventions received by performers (A) and how often the demonstration video was referred to (B) while the triads were practicing BMV or IVC. Collectively, students received more interventions from their triad peers while practicing IVC, compared to BMV, and this trend was statistically significant based on Mann-Whitney U test. Moreover, there were significantly more students who referred to the video while practicing IVC in comparison to BMV, according to Pearson’s Chi-square test.

**Table 1:** Students’ behavior while practicing BMV and IVC within a triad.

A. Number of interventions received			
		Bag mask ventilation (BMV)	Intravenous cannulation (IVC)
Number of students		104	107
Mean		1.2	3.6
Median		1	4
Std. Deviation		1.8	2.5
Minimum		0	0
Maximum		7	11
Shapiro-Wilk test		*p* < 0.001	*p* < 0.001
Mann-Whitney U test (2-tailed)		*p* = 7.30 × 10^−15^**	
B. Referring to the instructional video			
		Bag mask ventilation (BMV)	Intravenous cannulation (IVC)
Number of students		104	107
Referred to instructional video	Yes	2	15	
	No	102	92
Pearson Chi-square test (2-tailed)		*p* = 0.001**	

** Difference is significant at the 0.01 level (2-tailed).

Table 2 shows the total scores awarded for students’ performance of BMV and IVC by two independent assessors. For both skills, the scores given by both assessors were non-parametric as shown by Shapiro-Wilk test. Moreover, assessor 2 generally awarded lower scores than assessor 1, and Wilcoxon signed-rank test confirmed that there was significant difference between the assessors in their scoring of students’ performance of BMV and IVC. However, there was a significant correlation between assessors 1 and 2 when they scored BMV and IVC performance, as shown by Kendall’s tau-b, suggesting that they were consistent in terms of overall pattern of scoring.

**Table 2:** Scores awarded for the performance of BMV and IVC by two independent assessors.

		Bag mask ventilation (BMV)		Intravenous cannulation (IVC)	
		Assessor 1	Assessor 2	Assessor 1	Assessor 2
Number of students		158	159	162	162
Mean		8.3	5.7	15.7	12.7
Standard deviation		1.6	1.6	1.5	1.8
Minimum		2.8	0.0	7.4	7.5
Maximum		10.2	9.0	17.8	16.4
Percentiles	25	7.0	4.6	14.9	11.6
	50	8.7	5.9	15.9	12.9
	75	9.7	6.9	16.7	14.1
Total scores attainable		10.2		17.8	
Shapiro-Wilk test		*p* = 1.16 x 10^−7^**	*p* = 0.00245**	*p* = 6.88 x 10^−9^**	*p* = 0.000292**
Number of students scored below 50%		4	57	1	7
% of students scored below 50%		2.5%	36%	<1%	4.3%
Kendall’s tau-b (2-tailed)		tau-b = 0.539; *p* = 3.55 x 10^−22^**		tau-b = 0.250; *p* = 3 x 10^−6^**	
Wilcoxon signed-rank test (2-tailed)		*p* = 1.18 x 10^−27^**		*p* = 4.03 x 10^−27^**	

**Significant at the level of 0.01 (2-tailed).

### Influence of video-watching behavior and grouping on skill performance

The average values between assessor 1 and assessor 2’s scores for BMV and IVC performance were computed. Table 3 shows the average scores attained by (i) students who claimed to have watched the demonstration video before class versus those who did not claim so, and (ii) students of BMV^1st^ group versus those of IVC^1st^ group. Students who claimed to have watched the demonstration video for BMV before class collectively attained a higher average score for BMV performance. Similarly, students of BMV^1st^ group attained a higher average score in BMV than those of IVC^1st^ group. These differences in mean score were statistically significant based on Mann-Whitney U test. However, for IVC performance, there was no significant difference in the average scores attained by (i) students who claimed to have watched the demonstration video before class versus those who did not claim so, and (ii) students of BMV^1st^ group versus those of IVC^1st^ group.

**Table 3:** Skill performance across different groups.

A. Bag mask ventilation (BMV)				
	(i) Claimed to have watched the video before class		(ii) Group sequence	
	Yes^1^	No^2^	BMV^1st^	IVC^1st^
Mean	7.8	6.3	7.8	6.0
Standard deviation	1.0	1.7	1.0	1.6
Number of students	71	88	85	74
Mann-Whitney U test (2-tailed)	*p* = 3.25 ×10^−9^**		*p* = 1.29 ×10^−12^**	
B. Intravenous cannulation (IVC)				
	(i) Claimed to have watched the video before class		(ii) Group sequence	
	Yes^3^	No^4^	BMV^1st^	IVC^1st^
Mean	14.3	14.1	14.3	14.1
Standard deviation	1.3	1.4	1.3	1.4
Number of students	58	104	84	78
Mann-Whitney U test (2-tailed)	*p* = 0.295		*p* = 0.669	

^1^BMV^1st^ students who answered “Yes” for question 1 in the survey.

^2^IVC^1st^ students + BMV^1st^ students who answered “No” or did not answer question 1.

^3^IVC^1st^ students who answered “Yes” for question 1 in the survey.

^4^IVC^1st^ students + BMV^1st^ students who answered “No” or did not answer question 1.

**Difference is significant at the level of 0.01 (2-tailed).

Table 4 shows the number of interventions received by (i) students who claimed to have watched the demonstration video before class versus those who did not claim so, and (ii) students of BMV^1st^ group versus those of IVC^1st^ group. Students who claimed to have watched the demonstration video for IVC received less interventions from their triad peers. Similarly, students of BMV^1st^ group on average received more interventions than those of IVC^1st^ group. These differences in the mean number of interventions were statistically significant based on Mann-Whitney U test (*p* < 0.05). The correlation between the number of interventions received and skill performance was determined by Kendall’s tau-b, and there were no statistically significant correlations (BMV: tau-b = 0.141, *p* = 0.065, *n* = 100; IVC: tau-b = -0.046, *p* = 0.512, *n* = 107). Therefore, receiving less intervention from peers do not equal better skill performance.

**Table 4:** Students’ behavior within the triad across different groups.

A. Bag mask ventilation				
	(i) Claimed to have watched the video before class		(ii) Group sequence	
	Yes^3^	No^4^	BMV^1st^	IVC^1st^
No. of interventions received				
Mean	1.8	1.0	1.9	0.8
Standard deviation	2.4	1.5	2.5	1.2
Number of students	30	74	38	66
Mann-Whitney U test (2-tailed)	*p* = 0.290		*p* = 0.195	
Referred to instructional video				
Yes	0	2	0	2
No	30	72	38	64
Number of students	30	74	38	66
Pearson Chi-square test (2-tailed)	*p* = 0.363		*p* = 0.279	
B. Intravenous cannulation				
	(i) Claimed to have watched the video before class		(ii) Group sequence	
	Yes^3^	No^4^	BMV^1st^	IVC^1st^
No. of intervention received				
Mean	2.9	4.3	4.9	3.0
Standard deviation	1.8	2.9	3.1	1.8
Number of students	51	56	38	69
Mann-Whitney U test (2-tailed)	*p* = 0.017*		*p* = 0.002**	
Referred to instructional video				
Yes	6	9	6	9
No	45	47	32	60
Number of students	51	56	38	69
Pearson Chi-square test (2-tailed)	*p* = 0.522		*p* = 0.695	

^1^BMV^1st^ students who answered “Yes” for question 1 in the survey.

^2^IVC^1st^ students + BMV^1st^ students who answered “No” or did not answer question 1.

^3^IVC^1st^ students who answered “Yes” for question 1 in the survey.

^4^IVC^1st^ students + BMV^1st^ students who answered “No” or did not answer question 1.

*Difference is significant at the level of 0.05 (2-tailed).

**Difference is significant at the level of 0.01 (2-tailed).

### Students’ perception

Table 5 summarizes the results and item-total correlations for questions 2 to 11 of the post-class questionnaire. Between these 10 items, Cronbach’s alpha value was 0.844, and all item-total correlations were greater than 0.4, hence the internal reliability of the questionnaire was acceptable.

**Table 5:** Summary of post-lesson questionnaire.

	Questions	Means± S.D.	Item-total correlations	Kendall’s tau-b (with Q11)
Q2.	You found watching the instructional video out of class helpful.	5.0 ± 0.8	0.459	0.166*
	(Strong disagree = 1; strongly agree = 6)	(N = 146)		
Q3.	You found watching the instructional video in class with peer interaction helpful.	4.8 ± 0.1	0.483	0.295**
	(Strong disagree = 1; strongly agree = 6)	(N = 164)		
Q4.	The content of the instructional videos for IV cannulation was useful for acquiring the skill.	5.3 ± 0.8	0.544	0.311**
	(Strong disagree = 1; strongly agree = 6)	(N = 164)		
Q5.	The content of the instructional video for bag mask ventilation was useful for acquiring the skill.	5.2 ± 0.8	0.577	0.228**
	(Strong disagree = 1; strongly agree = 6)	(N = 164)		
Q6.	You found participating as the coach useful for acquiring the skill.	4.9 ± 1.0	0.540	0.403**
	(Strong disagree = 1; strongly agree = 6)	(N = 164)		
Q7.	You found participating as the monitor useful for acquiring the skill.	4.5 ± 1.1	0.570	0.406**
	(Strong disagree = 1; strongly agree = 6)	(N = 163)		
Q8	You found rotation in the different roles useful for acquiring the skill.	4.9 ± 0.9	0.637	0.442**
	(Strong disagree = 1; strongly agree = 6)	(N = 164)		
Q9.	The content of the supplementary information for the IV cannulation was useful.	5.0 ± 0.8	0.575	0.336**
	(Strong disagree = 1; strongly agree = 6)	(N = 161)		
Q10.	The content of the supplementary information for bag mask ventilation was useful.	4.9 ± 0.9	0.604	0.296**
	(Strong disagree = 1; strongly agree = 6)	(N = 161)		
Q11.	Please compare existing approaches to this approach for learning clinical skills. The teaching approach of this lesson is:	4.2 ± 0.7	0.466	N/A
	(Much worse = 1; much better = 5)	(N = 159)		

** Correlation is significant at the 0.01 level (2-tailed).

* Correlation is significant at the 0.05 level (2-tailed).

**Fig 1:**
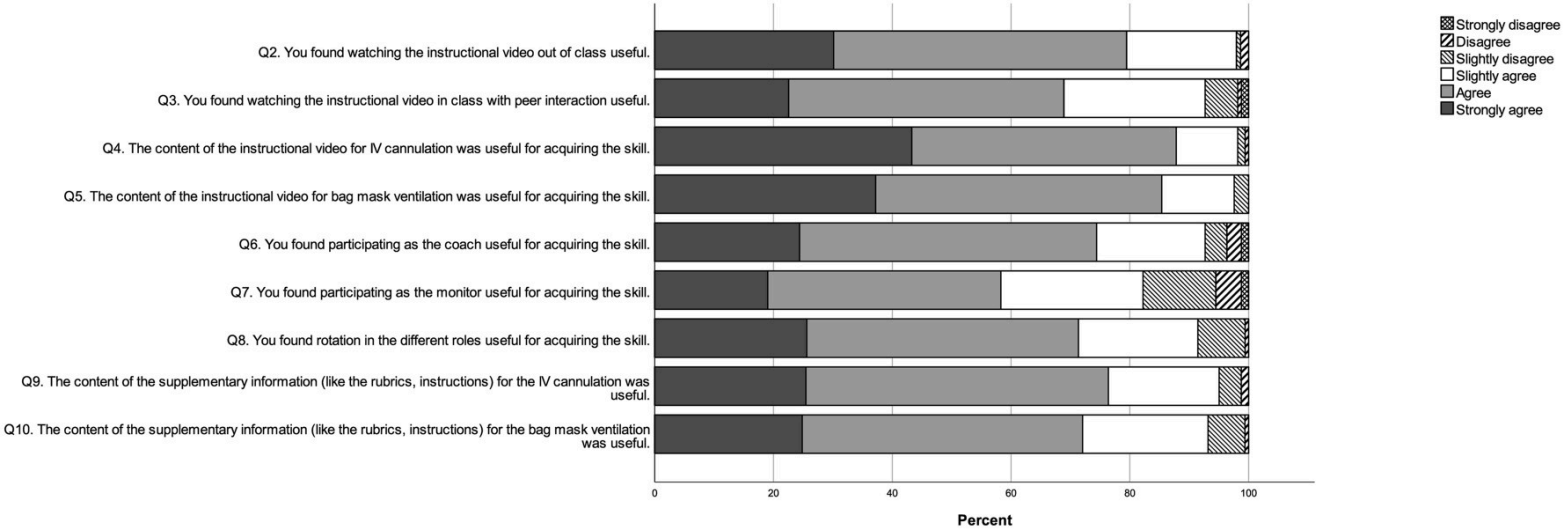
Divided bar chart showing the frequency of responses (% of total) to questions 2 to 10 of the post-class evaluation questionnaire.

As shown in Fig 1, we found that most students agreed or strongly agreed that it was useful to watch the demonstration videos out of class (79.4%) or in class (68.9%). Most students agreed or strongly agreed that the demonstration videos were useful for skill acquisition (IVC: 87.8%; BMV: 85.4%). Moreover, most students agreed or strongly agreed that supplementary information like the rubrics or written instructions were useful (IVC: 76.4%; BMV: 72.0%). For in-class arrangements, 74.4% agreed or strongly agreed that participating as the coach within a triad was useful for skill acquisition, while 58.3% agreed or strongly agreed that participating as the monitor was useful. There were 71.3% who agreed or strongly agreed that rotating different roles within a triad was useful for skill acquisition.

**Fig 2:**
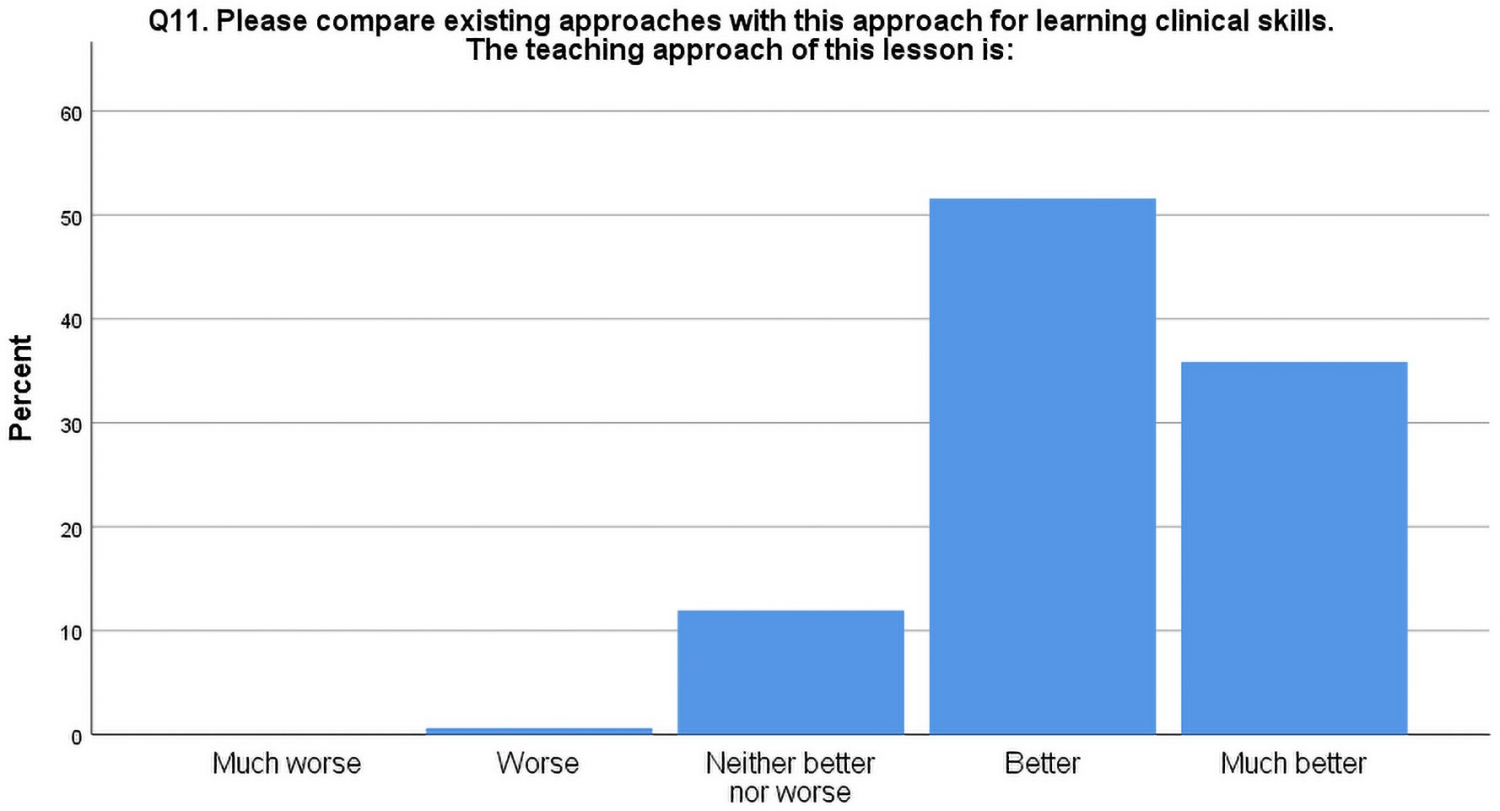
Bar chart percentage showing the frequency of responses (% of total) to question 11 of the post-class evaluation questionnaire.

As shown in Fig 2, when asked to compare this new approach of clinical skills teaching with the existing approach, the majority of students thought the new approach was better. As shown in Table 5, based on Kendall’s tau-b, there were statistically significant positive correlations (tau-b > 0.4; p < 0.01) between students’ perceived superiority of this new approach over traditional approach (Q11) and the perceived usefulness of being a “coach” (Q6), being a “monitor” (Q7) and rotating between different roles within a triad (Q8).

## Discussion

In this study, we trialed a flipped classroom, same-level peer-assisted learning model for teaching clinical procedural skills. We found that nearly 80% of our students were compliant and watched the assigned demonstration videos before class. A small proportion of students referred to the demonstration videos during trial practices; and interestingly, a higher number of students referred to the demonstration video during triad practice for IVC compared to BMV. This trend was consistent with our expectation, because IVC is a more complex procedure than BMV.

As we evaluated the influence of pre-class video watching on performance scores, students who claimed to have watched the demonstration video for BMV before class achieved higher scores in BMV performance compared to those who only watched during the class; and this trend was not observed for IVC. Interestingly, students who claimed to have watched the IVC demonstration video before class received less interventions from their peers while practicing IVC. While a lower number of interventions received could be interpreted as having made less mistakes during the practice, it does not imply better skill performance at the end, and our statistical analysis showed no significant correlation between the number of interventions received during triad practice and performance score. Of note, there is currently a lack of evidence demonstrating any relationship between the timing of video watching and clinical skill acquisition in the literature; and based on the findings of this study, the relationship between the timing of video watching and skill acquisition remains inconclusive.

As mentioned previously, IVC is a more complex procedure compared to BMV, and it is plausible that this difference in complexity could account for the differing trends for BMV and IVC. Previous studies on the impact of demonstration video on clinical skills learning also showed heterogenous outcome, where some studies reported that demonstration video enhances skill performance compared to traditional approaches, while others reported no significance difference [24, 25]. However, these studies did not determine if the complexity of procedures influences the relative merits of using demonstration videos.

Based on the post-class questionnaire, most of our students agreed that watching the demonstration videos for BMV and IVC were useful for their learning, providing the proof of principle that clinical skills demonstration videos are generally well received by medical students, as established by previous studies [23, 28]. Most of our students also indicated in the questionnaire that they preferred the new approach of clinical skill teaching and perceived that being a “monitor” and “coach” and rotating between different roles within a triad were useful for their skill acquisition. These findings are congruent with another study which compared dyad practice with individual practice for clinical skill learning [19]. However, this satisfaction could also be due to novelty effect, as it is established that humans tend to have heightened engagement when they encounter a novel technology [41, 42].

With regards to inter-assessor consistency, while the overall pattern of scoring by assessors 1 and 2 were consistent, there was significant difference in the scores awarded between the assessors. We noted that the assessors had different interpretation of the marking rubric and expectations of the standards required. For instance, one of them only marked a step as “competent” if the performer articulated aloud what they were doing, and successfully performed every component of that step. To ensure consistency between the assessors, standardization through a training session or meeting is required, as practiced by previous studies [19, 43].

The assessors also noted that students of the same triad tend to make similar mistakes when performing the procedures. While we did not investigate this phenomenon in further details, we postulate that students could have been influenced by their peers while they were observing (e.g., when acting as the coach, the monitor, or recording video for their peers etc.). This highlights a weakness of our current lesson design, as our students were not immediately corrected after they observed a flawed performance as per good practices reported in the literature [21]. The tutors also shared anecdotes about some monitors who gave feedback or hints to the performer when they were not supposed to do so, suggesting that students’ compliance impacts the execution of this mode of clinical skill training.

There were some limitations in this study. We were not able to determine how triad practice compares to dyad practice or individual practice for clinical skill learning, due to the lack of control groups in this study. While it was shown that triad practice was non-inferior to dyad and individual practices for non-clinical procedures [20], there is currently no evidence suggesting that this principle also applies to clinical skill learning. Furthermore, this study only assessed students’ performance of the procedures immediately after triad practice, and students’ retention of the procedural skills in longer term and their ability to perform the procedures in a different context were not assessed. Based on the literature, successful performance of a clinical procedure in class do not necessarily indicate a learner’s retention of that skill and ability to apply it in other contexts [44, 45], therefore further investigations on learners’ retention of clinical procedural skill and skill transfer are warranted.

## Conclusions

In this study, we introduced a flipped classroom, same-level peer-assisted learning approach to teach clinical procedural skills to medical students. It involves having students watch a demonstration video developed by a clinical expert before class, followed by self-directed triad practices, where each member rotates through the roles of “performer”, “coach” and “monitor”. Most of our students were compliant with watching the assigned demonstration videos before class, as well as fulfilling their assigned roles in the triad.

Interestingly, a higher number of students referred to the demonstration video while practicing IVC compared to BMV. As we evaluated the influence of pre-class video watching on skill performance, students who claimed to have watched the demonstration video for BMV before class achieved higher score in BMV performance. On the other hand, students who claimed to have watched the IVC demonstration video before class received less intervention from their peers while practicing IVC, but this was not correlated with better or worse IVC performance. As such, we could not ascertain whether the timing when the demonstration video is watched would influence skill acquisition. Most of our students preferred this new approach of clinical procedural skill teaching and perceived that being a “monitor” and “coach” and rotating between different roles within a triad were useful for their skill acquisition.

Given the capacity constraints in teaching hospitals and medical students’ underexposure to clinical procedural skills in medical schools worldwide, this model is potentially an effective way to address the current challenges and improve the efficiency of clinical procedural skill teaching in medical schools.

## Supporting information

S1 File Handout for bag mask ventilation. (DOCX)

S2 File Handout for intravenous cannulation. (DOCX)

S3 File Rubric for bag mask ventilation. (DOCX)

S4 File Rubric for intravenous ventilation. (DOCX)

S5 File Post-class evaluation questionnaire. (DOCX)

S1 Dataset (XLSX)
