# Supplementary material for: A flipped classroom, same-level peer-assisted learning approach to clinical skill teaching for medical students
Source: PLoS One. 2021 Oct 22;16(10):e0258926. doi: 10.1371/journal.pone.0258926 (PMC8535182; doi:10.1371/journal.pone.0258926)
Supplement: S1 File — (DOCX) [file pone.0258926.s001.docx]

**BAG MASK VENTILATION**

------------------------------------------------------------------------

***Gather Equipment***

------------------------------------------------------------------------

- A self-inflating bag device
  - Self-inflating bag
  - An outlet valve assembly
  - Oxygen reservoir bag
  - Oxygen tubing
  - An inlet valve assembly
    - oxygen inlet port
    - connecting port for oxygen reservoir bag
- Airway adjuncts
  - OPA, NPA
- Facemask
  - 2 different sizes

------------------------------------------------------------------------

***Identifying the component and function***

-----------------------------------------------------------------------

- Self-inflating bag:
  - Draws air in from the atmosphere and or an oxygen source and displaces the
- An outlet valve assembly
  - Connects the bag to the mask
  - Contains fishmouth type one way valve that prevents rebreathing of expired air from patient
- Oxygen reservoir bag
  - Provides a source of oxygen that can be drawn upon during inspiration where the flow rate exceed that which could be supplied external oxygen source (wall or tank)
- Oxygen tubing
  - Connects the bag to external oxygen source
- An inlet valve assembly
  - Connects additional oxygen supply to the bag
    - Oxygen inlet port to oxygen source
    - Connecting port for oxygen reservoir bag

-----------------------------------------------------------------------

***Selecting the right mask size***

-----------------------------------------------------------------------

- Mask should cover the bridge of the nose and over the mouth just above the chin

-----------------------------------------------------------------------

***Assembling the self inflating bag and mask***

-----------------------------------------------------------------------

- Facemask and must be closest to the patient’s face
- Connect fishmouth end of outlet valve assembly to the mask
- Connect the remaining end of the outlet valve assembly to the smaller non-threaded opening of the bag
- Connect the inlet valve assembly to the larger threaded end of the bag.
  - Ports must face outwards
- Connect the reservoir bag to the larger port
- Connect the oxygen tubing to the smaller port

-----------------------------------------------------------------------

***Checking the equipment***

-----------------------------------------------------------------------

- Inspect the function of the one way valve
  - Opens and closes with compression and reinflation of the bag.
- Check for leak and ability to generate a positive pressure
  - occluding the outlet valve assembly, squeeze bag and check for resistance

-----------------------------------------------------------------------

***Opening the patient’s airway***

-----------------------------------------------------------------------

- Basic airway manoeuvres should be applied during ventilation
  - head tilt, chin lift +/- jaw thrust

-----------------------------------------------------------------------

***Holding the mask***

-----------------------------------------------------------------------

- Place thumb & index finger around the hole of the mask to form a “C”
- Place the facemask over the nose and mouth of the patient and apply gentle and perpendicular downward pressure on the mask to establish a seal
- Place the remaining fingers under the mandible to form an “E”
- Anchor the hand to the face by pulling the face towards the mask.
- Keep your fingertips over only the bony part of the mandible
  - Avoid pressing on the floor of the mouth.
- Check for chin lift, head tilt +/- jaw thrust
- Recognise whether a 2 handed technique is indicated
  - If used, second person should be requested to squeeze the bag

-----------------------------------------------------------------------

***Squeezing the bag***

-----------------------------------------------------------------------

- Hold the bag securely from either above or below to avoid dropping the bag
- Squeeze the bag gently.
- Deliver enough air to see the chest rise.
- Release the bag and allow it to fill passively with air.
- Squeeze again at a rate of between 10 to 12 breaths per minute.

-----------------------------------------------------------------------

***Assessing the adequacy of ventilation***

----------------------------------------------------------------------

- Observe the patient’s chest movements
- (or ask a colleague to auscultate the chest )
- If chest movement not obvious, need to exclude one of the following issues
  - Adequacy of the facemask seal
  - Integrity of the equipment
  - Patency of airway

-----------------------------------------------------------------------

***Adequacy of the facemask seal***

-----------------------------------------------------------------------

- Look, feel or listen for air leak near the bridge of the nose, or the side of the mask and correct accordingly
- Check for position of mask again if oropharyngeal airway inserted

-----------------------------------------------------------------------

***Check integrity of the equipment***

-----------------------------------------------------------------------

- Inspect the entire device for disconnection, especially with the outlet valve assembly

-----------------------------------------------------------------------

***Check the patency of the upper airway***

-----------------------------------------------------------------------

- Check that you are applying the basic airway manoeuvres correctly.
- Check for foreign body if not already done so
- If satisfied with the above use an airway adjunct and reassess

-----------------------------------------------------------------------
