# Supplementary material for: A flipped classroom, same-level peer-assisted learning approach to clinical skill teaching for medical students
Source: PLoS One. 2021 Oct 22;16(10):e0258926. doi: 10.1371/journal.pone.0258926 (PMC8535182; doi:10.1371/journal.pone.0258926)
Supplement: S2 File — (DOCX) [file pone.0258926.s002.docx]

**INTRAVENOUS CANNULATION**

------------------------------------------------------------------------

***Gathering equipment***

------------------------------------------------------------------------

- Hand sanitiser
- Gloves
- A tourniquet
- A Cannula
- Dressing
- A Sharps bin
- Syringe with saline
- A Connector (or a 3 way tap)
- Prime the connector with saline
  - Ensure there are no air bubbles

------------------------------------------------------------------------

***Patient preparation***

------------------------------------------------------------------------

Patient in a comfortable position

Apply the tourniquet firmly

- Above point of entry
- Secure not come loose
- No discomfort

------------------------------------------------------------------------

***Hand hygiene***

-----------------------------------------------------------------------

- disinfect your hands
- put on gloves

------------------------------------------------------------------------

***Vein distension***

------------------------------------------------------------------------

- Open and close his hand
- Tap on it gently.
- Put arm in a dependent position if necessary
- Ensure vein can accommodate entire cannula.

------------------------------------------------------------------------

***Skin Cleansing***

------------------------------------------------------------------------

- Clean the vein with an antiseptic wipe (proximal to distal)

------------------------------------------------------------------------

***Holding the cannula***

------------------------------------------------------------------------

- Hold cannula so flashback chamber see seen from above

------------------------------------------------------------------------

***Immobilising the vein***

------------------------------------------------------------------------

- Apply slight tension on the skin proximal to entry point
- Avoid excessive tension causing vein collapse

------------------------------------------------------------------------

***Needle insertion***

------------------------------------------------------------------------

- Insert with needle the bevel up
- Approach at an angle of between 10 to 30 degrees.
- Puncture the skin smoothly
- Watching for blood to enter the flashback chamber
- Once flashback occurs, swiftly reduce the angle of the cannula and advance the needle 2 mm further
- Ensure blood still flowing into the chamber

------------------------------------------------------------------------

***Cannula advancement***

------------------------------------------------------------------------

- Stabilise the needle
- Slide the cannula into the lumen of the vein all the way to the hub.
  - Do NOT pull the needle back

------------------------------------------------------------------------

***Tourniquet release***

------------------------------------------------------------------------

- Apply gentle proximal to the tip of the cannula
- Release the tourniquet

------------------------------------------------------------------------

***Needle removal and disposal***

------------------------------------------------------------------------

- Gently take the needle out without removing the cannula itself.
- Ensure the safety feature of the cannula is engaged as appropriate.
- Discard the needle into the sharps bin

------------------------------------------------------------------------

***Applying the connector***

------------------------------------------------------------------------

- Keep applying pressure with the finger
- Apply the connector to the hub of the cannula
  - ensure it is screwed on properly.
- Inject a small volume of saline to check for extravasation along the course of the vein.

------------------------------------------------------------------------

***Apply dressing***

------------------------------------------------------------------------

- Cover the puncture site, the hub as well as a part of the connecting device with dressing
- Ensure good contact with the cannula and patient’s skin
- Avoid excessive pressure with the dressing
- Anchor the connector to the skin by using an extra bit of tape

------------------------------------------------------------------------

***Cleaning up***

------------------------------------------------------------------------

- Dispose all your wastes appropriately
