# Supplementary material for: A flipped classroom, same-level peer-assisted learning approach to clinical skill teaching for medical students
Source: PLoS One. 2021 Oct 22;16(10):e0258926. doi: 10.1371/journal.pone.0258926 (PMC8535182; doi:10.1371/journal.pone.0258926)
Supplement: S3 File — (DOCX) [file pone.0258926.s003.docx]

Appendix 3 Bag Mask Ventilation Rubric

| ***General description of level of competencies*** | ***Competent***  Generally able to complete the task without prompting | ***Needs improvement***  Omits a non-critical aspect of the step but respond appropriately to non-specific prompting  (eg have you missed out on anything at this stage) | ***Incompetent***  Omits a critical aspect of the step and does not respond appropriately to specific prompting  (eg did you omit to connect a part to the outlet assembly so the device can fit on the patient’s face?) |
| --- | --- | --- | --- |
| **Gather equipment** | Gathered complete list of equipment   - Bag - Outlet valve assembly - Inlet valve assembly - Oxygen reservoir bag - Oxygen tubing - Mask - Nasopharyngeal Airway - Oropharyngeal airway | Omits several items (inlet valve assembly and associated parts and or airway adjuncts) but may still be able to deliver air to patient with items gathered | Omits one of more of the critical components   - Mask - Bag - Outlet valve assembly |
| **Identifying the components** | Able to name all the parts and their respective function | Unable to recall name and or function of one to two parts | Unable to recall name and or function of three or more parts |
| **Selecting the right size mask** | Able to describe and demonstrate how to correctly size a mask on a patient’s face by pointing out the relevant landmarks for the mask to cover | Selects the correct size only after prompting or unable to describe why the size maybe appropriate | Select the inappropriate mask and unable to recognise the mistake even after specific prompting |
| **Assembling the equipment** | Correctly assembles the whole device | Commit errors but responded to non-specific prompting | Commit errors but failed to respond to non-specific prompting |
| **Checking the equipment** | Thoroughly checked the integrity of the system at each connection point and checks that the bag can generate positive pressure by feeling for resistance with the opening occluded | Missed checking or failed to articulate one component of the system check  eg connecting to the oxygen supply | Failed to notice a critical point of dysfunction in the device  eg, no mask attached |
| **Opening the patient’s airway** | Appropriately applying head tilt, chin lift +/- jaw thrust | Respond to non-specific prompting | Does not respond to non-specific prompting and performs BVM without paying attention to basic airway manoeuvres |
| **Holding the mask**  Squeezing the bag also has to be performed here to demonstrate correct mask holding but is scored differently | Correctly performs the E-C technique of holding the mask in the correct position without a leak; recognises that a two-handed technique maybe needed | Performs one or more elements of the mask holding incorrectly but responds to non-specific prompting  ☐ leaves gap between mask and face  ☐ incorrect placement of mask over nose or mouth  ☐ pressing the floor of the mouth with fingers | Performs one or more elements of the mask holding incorrectly but does NOT respond to prompting leading to audible leak and or minimal chest movement upon ventilating attempt  ☐ leaves gap between mask and face  ☐ incorrect placement of mask over nose or mouth  ☐ pressing the floor of the mouth with fingers |
| **Squeezing the bag** | Able to sustain all of the following for greater than 60 seconds  ☐ consistent visible chest rise (8 or more good breaths out of every 10)  ☐ Holds the bag securely and not dropping it  ☐ No wayward force in the device that leads to disconnection of anyone of the parts  ☐ squeezes the bag at correct rate | Fails at 1 or more of the following but able to correct deficiencies with prompting  ☐ consistent visible chest rise (8 or more good breaths out of every 10)  ☐ Holds the bag securely and not dropping it  ☐ No wayward force in the device that leads to disconnection of anyone of the parts  ☐ squeezes the bag at correct rate | Unable to achieve chest rise for more than half of the ventilator attempts  Fails at 2 or more of the following  ☐ Holds the bag securely and not dropping it  ☐ No wayward force in the device that leads to disconnection of anyone of the parts  ☐ squeezes the bag at correct rate |
| **Assessing the adequacy of ventilation** | Recognises inadequacies and automatically corrects it without prompting  Able to describe the common technical causes of inadequate ventilation | Recognises inadequacies and corrects it only after non-specific prompting | Unable to recognise inadequacies in ventilation even after specific prompting |
