# Supplementary material for: A flipped classroom, same-level peer-assisted learning approach to clinical skill teaching for medical students
Source: PLoS One. 2021 Oct 22;16(10):e0258926. doi: 10.1371/journal.pone.0258926 (PMC8535182; doi:10.1371/journal.pone.0258926)
Supplement: S4 File — (DOCX) [file pone.0258926.s004.docx]

Appendix 4 Intravenous Cannulation Rubric

|  |  | Competent | Needs improvement | Incompetent |
| --- | --- | --- | --- | --- |
| Gather equipment | Hand sanitiser  Gloves  Tourniquet  Cannula  Connector  Dressing  Syringe  Saline  Sharps bin | Gathered complete list of equipment gathered  Checked function and or used by date  Prepared for immediate access and use | Omitting some of the components  Failed to check some of the components but does so after non-specific prompting | Omit components that causes major disruption, failure to complete the procedure or cause mishaps such as blood spillage or dislodgement of the cannula |
| Apply tourniquet | Adequate distance above the target point of entry (15cm)  Ensure no skin is pinched by tourniquet  Ensure the tourniquet is functioning | Apply in suitable position, appropriate tension and without causing excessive discomfort | Application requiring adjustment in terms of position, tension or the need to relieve patient of pain from tourniquet | Not applying before cannulation  Cause significant pain to patient without realising it |
| Hand hygiene and gloves | Hand washing or alcohol rub  Apply gloves of correct size and of full integrity | Appropriate disinfection technique and gloves applied correctly, and integrity checked | Omit either hand washing or gloving but does so with non-specific prompting | Omit one or another step requiring specific reminder to do so |
| Manoeuvres to distend veins | Ask patient either exercise the hand and or tapping on vein | Ask patient to perform either action without prompting | Ask patient to do with non-specific prompting | Do not ask patient to do anything to facilitate vein distension after non-specific prompting |
| Vein selection and skin cleansing | Vein should be of adequate length and calibre to accommodate cannula  Area of entry should be cleaned with alcohol wipe and wait for 30 seconds  Do not touch site after cleaning | Select a vein that can accommodate the proposed size cannula  Clean the correct area and waited for the appropriate length of time before attempting insertion | Selects a vein that may be borderline in accommodating the cannula  Need prompting to clean the area | Select a vein that is unlikely to accommodate the cannula  Did not clean skin before proceeding |
| Handling of cannula | Hold cannula alongside the flashback chamber to enable adequate visualisation | Securely holding the cannula that enable visualization of the flashback chamber | Needed non-specific prompting to reposition the fingers | Holding the cannula that obscures the flashback chamber or is unstable for insertion |
| Immobilization of target vein | Pulling gently on skin distal to entry point with non-inserting hand | Apply traction at an appropriate location and effectively immobilizes the vein | Requires non-specific prompting or pulling skin in a position that obstructs the insertion of cannula | Requires specific prompting of the need to stabilise vein |
| Needle insertion | Bevel up  10 to 30  Puncture skin in a smooth fashion  Watch for flashback of blood | Approaches vein at the appropriate angle  Puncture skin in a single smooth action  Stops forward motion at the first indication of flashback | Require prompting to adjust the angle of entry of needle  Double puncture of vein but able to salvage  More than one attempt to obtain flashback | Unable to obtain flashback (missed the vein)  Double puncture the veins and unable to salvage |
| Cannula advancement | Ensures the intraluminal location of the tip of the cannula  Stabilise the stylette and advances the hub until it reaches the skin | Reduce the angle of cannula,  advance the whole needle 1 to 2 mm in the direction of the vein  Stabilises the stylette and advance the hub until it reaches the skin | Advances cannula before ascertaining the tip is on the lumen but is able to recover | Fails to advance the cannula into the lumen of the vein |
| Tourniquet release | Release the tourniquet before removing the needle | Release the tourniquet without prompting | Prompting required | Did not release the tourniquet |
| Needle removal and disposal | Apply gentle pressure along the course of the vein proximal to the length of cannula  Gently removing the needle from the cannula without dispersing blood  Ensure the safety feature of the cannula is engaged  Disposal of needle into the sharps bin | Applies occluding pressure and remove needle in a smooth fashion | Needed prompting for one to 2 steps  Removes needle too briskly leading to some flicking of fluid or some movement of the cannula | No pressure applied  Removal of needle in a dangerous fashion  (harming others or themselves)  Not safely disposing sharp |
| Cannula attachment | Attach connecting device to the hub of cannula to prevent backflow of blood and allow injection and or intravenous fluid infusion | Without releasing the finger applying pressure to stop backflow, the connecting device is securely connected to the hub of cannula | Omit to occlude vein or let go of previous occlusion to stop backflow | Forgets to attach connector leading to backflow of blood |
| Check patency | Gentle injection of small amount of saline, checking for resistance, vibration from flow and evidence of extravasation (high resistance, significant pain or visible swelling) | Ensure the syringe is devoid of bubbles  Clean and securely connect to injection site  inject saline with a smooth gentle force while checking for flow and eliciting signs of extravasation: high resistance, pain or swelling | Require prompting to remove air bubbles or cleaning injection site;  or omitted checking for one of the signs of extravasation | Not connecting to injection site properly leading to spillage of fluid upon injection or not looking for any signs of extravasation |
| Apply dressing | Apply dressing of choice that anchors the cannula securing in place without applying excess pressure on the skin | Dressing applied securely covering the entry point with sufficient length and tension | Dressing not secured to patient after application | Omitted to apply dressing |
| Disposal of waste |  |  |  |  |
