# Supplementary material for: A flipped classroom, same-level peer-assisted learning approach to clinical skill teaching for medical students
Source: PLoS One. 2021 Oct 22;16(10):e0258926. doi: 10.1371/journal.pone.0258926 (PMC8535182; doi:10.1371/journal.pone.0258926)
Supplement: S5 File — (DOCX) [file pone.0258926.s005.docx]

Appendix 5 Students’ Questionnaire

1. Did you watch the video before class?
   - Yes
   - No
2. You found watching the instructional video out of class useful.

Strongly disagree 1 2 3 4 5 6 Strongly agree

1. You found watching the instructional video in class with peer interaction helpful.

Strongly disagree 1 2 3 4 5 6 Strongly agree

1. The content of the instructional video for IV cannulation was useful for acquiring the skill.

Strongly disagree 1 2 3 4 5 6 Strongly agree

1. The content of the instructional video for bag mask ventilation was useful for acquiring the skill.

Strongly disagree 1 2 3 4 5 6 Strongly agree

1. You found participating as the coach useful for acquiring the skill.

Strongly disagree 1 2 3 4 5 6 Strongly agree

1. You found participating as the monitor useful for acquiring the skill.

Strongly disagree 1 2 3 4 5 6 Strongly agree

1. You found rotation in the different roles useful for acquiring the skill.

Strongly disagree 1 2 3 4 5 6 Strongly agree

1. The content of the supplementary information (like the rubrics, instructions) for the IV cannulation was useful.

Strongly disagree 1 2 3 4 5 6 Strongly agree

1. The content of the supplementary information (like the rubrics, instructions) for the bag mask ventilation was useful.

Strongly disagree 1 2 3 4 5 6 Strongly agree

1. Please compare existing approaches with this approach for learning clinical skills:

The teaching approach of this lesson is:

Much worse 1 2 3 4 5 Much better
